# Setting Characteristics and High Compressive Strength of an Anti-washout, Injectable Calcium Phosphate Cement Combined with Thermosensitive Hydrogel

**DOI:** 10.3390/ma13245779

**Published:** 2020-12-17

**Authors:** Yao Xie, Jia Liu, Shu Cai, Xiaogang Bao, Qianqian Li, Guohua Xu

**Affiliations:** 1 Key Laboratory for Advanced Ceramics and Machining Technology of Ministry of Education, Tianjin University, Tianjin 300072, China; xieyao0726@tju.edu.cn (Y.X.); liqianqian@tju.edu.cn (Q.L.); 2 Department of Orthopedic Surgery, Spine Center, Naval Medical University, Shanghai 200003, China; 13506191593@163.com (J.L.); bxg1832178@smmu.edu.cn (X.B.)

**Keywords:** calcium phosphate cement, PLGA-PEG-PLGA, anti-washout property, compressive strength

## Abstract

In this work, a thermosensitive poly(D,L-lactide-co-glycolide)-poly(ethylene glycol)-poly(D,L-lactide-co-glycolide) (PLGA-PEG-PLGA) hydrogel was introduced into calcium phosphate cement (CPC) to enhance the anti-washout property of CPC. The effects of the hydrogel on the setting time, injectability, anti-washout property and compressive strength of CPC were thoroughly investigated. The results showed that the hydrogel significantly increased the injectability and anti-washout property of CPC, meanwhile maintained the setting time with an acceptable range. Moreover, the hydrogel improved the initial compressive strength of CPC. The composite cement with 20% *v*/*v* hydrogel in the liquid phase showed fine crystals of hydration product, a more compact microstructure and lower porosity compared with control CPC. The analysis of X-ray diffraction (XRD), infrared spectroscopy (FTIR) and X-ray photoelectron spectroscopy (XPS) indicated that suitable volume ratio of hydrogel (20% *v*/*v*) in the setting liquid of CPC could promote the formation of hydroxyapatite in the early hydration period. The degradation behavior of the cement was characterized by immersion tests in simulated body fluid. The hydrogel had no adverse effect on the degradation rate of CPC over the immersion period of 23 days. This study indicated that incorporating PLGA-PEG-PLGA hydrogel could be a promising strategy to reinforce the handing properties and initial compressive strength of calcium phosphate cement.

## 1. Introduction

Calcium phosphate cement (CPC) is generally considered as a promising bone substitution material in the fields of kyphoplasty or vertebroplasty due to its self-setting and biocompatibility [1]. CPC always consists of two phases, -solid particles and liquid, which, upon mixture with each other, form a moldable paste to fill the complex bone defect site and then undergo a hydration reaction, resulting in a strong material–bone interface [2,3]. The final cement product is hydroxyapatite (HA) or brushite phase, which resembles the inorganic components of human bone and thus provides a high level of biocompatibility and osteoconductivity [4,5,6,7].

Unfortunately, the CPC without any additives has a poor anti-washout property and tends to disintegrate when it contacts with body fluids at the surgical site, which may cause severe inflammatory reactions [8]. Decreasing the liquid-to-solid (L/P) ratio was useful to improve the anti-washout property of CPC, but a too low L/P ratio was not able to saturate the particles and result in the delay of setting rate [9]. Many efforts have been devoted to increasing the viscosity of the liquid phase of CPC by adding natural biopolymers, such as sodium polyacrylate (PAAS) [10], gelatin microspheres [11], guar gum [12], locust bean gum [13] and chondroitin sulfate [14] (see Table 1). However, the increase in the anti-washout property is always accompanied by a decline of compressive strength and an extension of setting time [15]. It has been widely reported that the initial mechanical property of CPC is also a critical issue that needs to be addressed, which decides whether the CPC could be strong enough to support bone regeneration [16,17]. Meanwhile, the CPC should have a suitable setting time to provide enough time for surgeons to perform the cement during surgery [18]. It is extremely urgent and necessary to find a new strategy to balance the setting time, injectability, anti-washout property and compressive strength of CPC.

Maazouz et al. [19] suggested that the preferred CPC paste is at a low viscosity when it is mixed and injected, and then rapidly turns to a high viscosity state once it fills the fracture site. The poly(D,L-lactide-co-glycolide)-poly(ethylene glycol)-poly(D,L-lactide-co-glycolide) (PLGA–PEG–PLGA) hydrogel is an aqueous solution containing PLGA-PEG-PLGA triblock copolymer, which consists of hydrophobic PLGA and hydrophilic PEG blocks [20]. This hydrogel is fluid at room temperature, and then rapidly converts to a viscous gel at a temperature close to body temperature via micelle aggregating and entangling [21]. It is usually used in the fields of drug delivery and tissue engineering because it is injectable, thermosensitive and biodegradable [22,23].

Herein, we aim to improve the anti-washout property of CPC by incorporating thermosensitive PLGA-PEG-PLGA hydrogel into the liquid phase of CPC and investigate the effects of PLGA-PEG-PLGA hydrogel on the setting characteristics and initial compressive strength of calcium phosphate cement.

## 2. Materials and Methods

### 2.1. Materials and Preparation

The solid particles consisted of α-Ca_3_(PO4)_2_ (α-TCP), Ca(H_2_PO_4_)_2_ H_2_O (Real& Lead Chemical Co., Ltd., Tianjin, China) and CaCO_3_ (Real& Lead Chemical Co., Ltd., Tianjin, China) at a mass ratio of 90:5:5. The α-TCP was synthesized by calcining a mixture of CaHPO_4_·2H_2_O (Chemart Co., Ltd., Tianjin, China)and CaCO_3_ (Real& Lead Chemical Co., Ltd., Tianjin, China) in a molar ratio of 2:1 at 1300 °C for 4 h. After air quenching, the calcinated α-TCP powder was ground in ethanol for 10 h at a rotation rate of 400 rpm and then dried at 65 °C. The liquid phase contained two parts: 4 wt.% disodium hydrogen phosphate (Na_2_HPO_4_) aqueous solution and PLGA-PEG-PLGA hydrogel. The PLGA-PEG-PLGA copolymer was synthesized through ring-opening copolymerization of D,L-lactide (LA) and glycolide (GA) using PEG as a macroinitiator and stannous octoate (Sn (Oct)_2_) as a catalyst [24]. LA, GA and Sn (Oct)_2_ were purchased from Sinopharm Chemical Reagent Co., Ltd., China. PEG (Mn = 1500 g mol^−1^) was purchased from Sigma-Aldrich, Germany. Briefly, 20.25 g PEG was heated at 125 °C under vacuum for 3 h. Then, 36.8 g LA, 9.9 g GA and 56 μL of Sn (Oct)_2_ toluene solution was added into the system and the mixture was vacuumized to remove the toluene. Subsequently, the mixture was reacted at 150 °C for 12 h to obtain the copolymer. The PLGA-PEG-PLGA hydrogel was prepared by dissolving the copolymer in deionized water at 4 °C.

**Figure 1:**
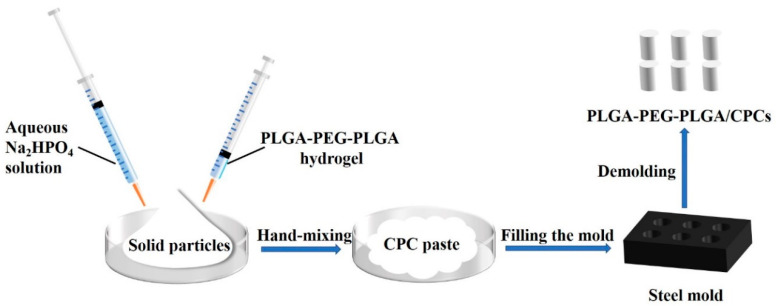
Schematic representation of the preparation process of PLGA-PEG-PLGA hydrogel/calcium phosphate cement composites (PLGA-PEG-PLGA/CPCs).

The preparation process of CPC and PLGA-PEG-PLGA hydrogel/calcium phosphate cement composites (PLGA-PEG-PLGA/CPCs) was shown in Figure 1. The disodium hydrogen phosphate (Na_2_HPO_4_) aqueous solution and PLGA-PEG-PLGA hydrogel was simultaneously added into the solid particles and manually mixed with each other to form injectable CPC pastes. The liquid-to-solid ratio was 0.5 mL/g. Then the fresh pastes were compacted into steel mold to obtain cylindrical samples with a size of 6 mm (diameter) × 12 mm (height). According to the different volume ratios of Na_2_HPO_4_ solution and hydrogel in the liquid phase, the CPCs were denoted as CPC (100% *v*/*v* Na_2_HPO_4_), CPC-10 (90% *v*/*v* Na_2_HPO_4_ and 10% *v*/*v* hydrogel), CPC-20 (80% *v*/*v* Na_2_HPO_4_ and 20% *v*/*v* hydrogel), CPC-30 (70% *v*/*v* Na_2_HPO_4_ and 30% *v*/*v* hydrogel), CPC-40 (60% *v*/*v* Na_2_HPO_4_ and 40% *v*/*v* hydrogel), as shown in Table 2.

### 2.2. Characterization

#### 2.2.1. Setting Time

The setting time of the CPC and PLGA-PEG-PLGA/CPCs was assessed by Vicat needle apparatus (Luda Experimental Instrument Co., Ltd., Shanghai, China) equipped with a light needle and a heavy needle [16,25]. The initial setting time was recorded when the light needle penetrated cement less than 1 mm. The final setting time was defined as the time when the heavy needle was not able not produce a complete circle indentation on the surface of cement. Each composite cement was measured six times.

#### 2.2.2. Injectability

A commercial syringe (inner aperture diameter of 2 mm) was used to measure the injectability of CPC and PLGA-PEG-PLGA/CPCs. Three grams of solid particles and 1.5 mL of setting liquid were poured into the syringe and blended with each other for 60 s to form the paste. Then, we extruded the paste at a crosshead speed of 15 mm/min until a maximum force of 250 N was reached [11]. The injectability of the cement was evaluated as the percentage of paste mass pushed out from the syringe [26]. Each composite paste was measured three times.

#### 2.2.3. Anti-washout Property

The anti-washout property of the CPC pastes was visual inspection of their disintegration under dynamic aqueous environment. The composite pastes were separately injected into simulated body fluid (SBF) at 37 °C and shaken at 60 rpm for 0 and 30 min respectively.

#### 2.2.4. Viscosity Test

The viscosity test of CPC and PLGA-PEG-PLGA/CPCs paste was performed on a rheometer (Waters, DHR-2, Beijing, China). The temperature of the test environment was controlled at 37 °C. 2 g of solid particle was mixed with the liquid phase at liquid to solid ratio of 0.5 mL/g for 2 min and then transferred to the parallel plate for the test.

#### 2.2.5. Compressive Strength Test

The compressive strength of the samples with a size of 6 mm (diameter) × 12 mm (height) was tested on a Universal Testing Machine (MTS, CMT4304, Shanghai, China) at a crosshead speed of 0.5 mm/min. The compressive strength of samples was obtained by the following equation:

Compressive strength (MPa) = 4F_max_/(πd^2^) (1)

where F_max_ was the maximum uniaxial compressive stress that the samples could understand before failure and d was the diameter of the samples. The compressive strength test was measured four times.

#### 2.2.6. Porosity

The porosity of the samples was measured according to the Archimedes principle [27]. After measuring the dried weight (W_0_) of samples, the samples were suspended in ethanol under vacuum for 2 h to make the ethanol infiltrate the pores of the samples. Then, we removed the ethanol on the surface of samples and measured the saturated weight (W_1_) of the samples. Finally, the suspended weight (W_2_) of the samples in ethanol was weighed. The porosity of the CPC was calculated by the following equation:

Porosity (%) = (W_1_ − W_0_)/ (W_1_ − W_2_) ×100% (2)

Five parallel samples were used for the porosity test.

#### 2.2.7. In Vitro Degradation

The as-prepared incompletely hydrated CPC samples were immersed in SBF solution at 37 °C with a surface area-to-volume 0.1 cm^2^/mL. The SBF solution was refreshed every two days. After a certain time, we took out the samples (initial weight W_0_) from the SBF solution and measured the pH value of SBF solution using pH meter. The hydrated samples were washed in deionized water and dried at 60 °C until constant weight (W_t_) was achieved. The weight loss percentage of each sample was calculated according to Equation (3):

Weight loss (%) = (W_0_ − W_t_)/W_0_ × 100% (3)

At least three parallel samples were used for the above test.

#### 2.2.8. Structural and Chemical Analysis

The phase compositions of hydrated cements were analyzed by X-ray diffraction (XRD; Bruker, D8 Advanced, Karlsruhe, Germany) using Cu Kα radiation (40 kV, 40 mA). The functional groups and chemical bonds of the hydrated cements were investigated using Fourier transform infrared spectroscopy (FTIR; Thermo Scientific, Nicolet Is10, Waltham, MA, USA) in the range of 4000–400 cm^−1^. The chemical compositions and chemical states of the cement surface were determined by X-ray photoelectron spectroscopy (XPS; Kratos, Axis Supra, Manchester, UK) using monochromatized Al Kα X-ray as excitation source. The measured binding energies were calibrated by the C 1s at 284.8 eV. The fracture surface of the cements after gold spraying was observed by scanning electron microscopy (SEM; Hitachi, S4800, Tokyo, Japan).

#### 2.2.9. Statistical Analysis

Data were presented as the means ± standard deviations. Statistical difference was analyzed by SPSS software using one-way analysis of variance (ANOVA) with Tukey’s test. *p* < 0.05 was considered as a significant level and *p* < 0.001 was considered to be a very significant level.

## 3. Result

### 3.1. Setting Time

**Figure 2:**
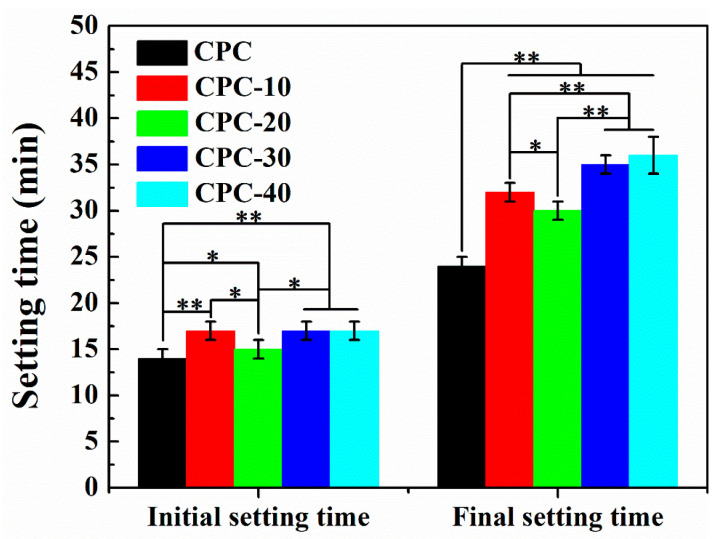
Setting time of control CPC and PLGA-PEG-PLGA/CPCs (n = 6). * indicates statistically significant differences between groups (** *p* < 0.001, * *p* < 0.05).

The initial and final setting time of PLGA-PEG-PLGA/CPCs and control CPC are shown in Figure 2. With the increase of hydrogel volume ratios from 10% *v*/*v* to 40% *v*/*v*, the initial and final setting time were prolonged to different degrees. Among them, the addition of 20% *v*/*v* hydrogel (sample CPC−20), slightly increased in both the initial and final setting time of CPC from 14 min and 24 min to 15 min and 30 min respectively, providing a wide time window for surgical procedures.

### 3.2. Injectability

**Figure 3:**
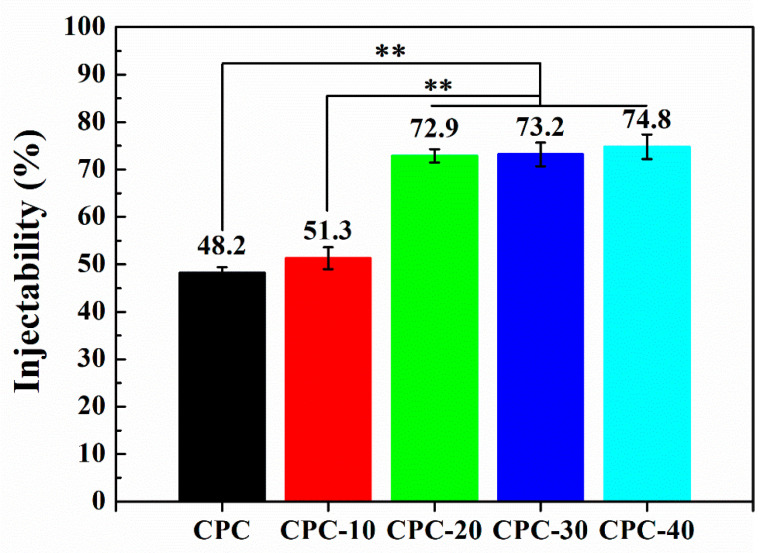
Injectability of CPCs containing different volume fractions of PLGA-PEG-PLGA hydrogel (n = 3, ** *p* < 0.001).

Figure 3 shows the influence of PLGA-PEG-PLGA hydrogel volume fraction on the injectability of CPC paste. It was obvious that the addition of hydrogel greatly improved the injectability of CPC paste. When the volume ratio of hydrogel was 20% *v*/*v* (sample CPC-20), the injectability reached to 72.9%, which was about 1.5 times higher than that of CPC. Further increasing the volume ratio of hydrogel, the injectability of composite cement pastes reached an upper limit at about 74.8% at the addition of 40% *v*/*v* hydrogel. In other words, the injectability of CPC paste did not increase any further even when the setting liquid was completely replaced by the hydrogel.

### 3.3. Antiwashout Property

**Figure 4:**
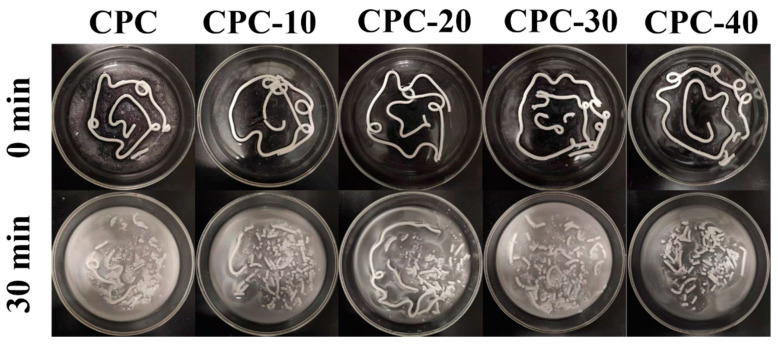
Optical images showing the anti-washout property of PLGA-PEG-PLGA/CPCs and control CPC. The pastes were injected into SBF solution at 37 °C and shaken for 0 min and 30 min.

Figure 4 represents the anti-washout property of CPC pastes containing various volume fractions of PLGA-PEG-PLGA hydrogel. When the paste was pushed out from the syringe and contacted with the SBF liquid for a short time, it was found that a few particles were separated from sample CPC, resulting in slightly turbid of SBF, whereas all the SBF solutions containing composite pastes with hydrogel were clear. After being shaken for 30 min, the tubelike shape pastes of sample CPC, CPC-10, CPC-30 and CPC-40 had a significant disintegration, but sample CPC-20 retained a longer tubelike shape paste than other samples.

### 3.4. Viscosity

**Figure 5:**
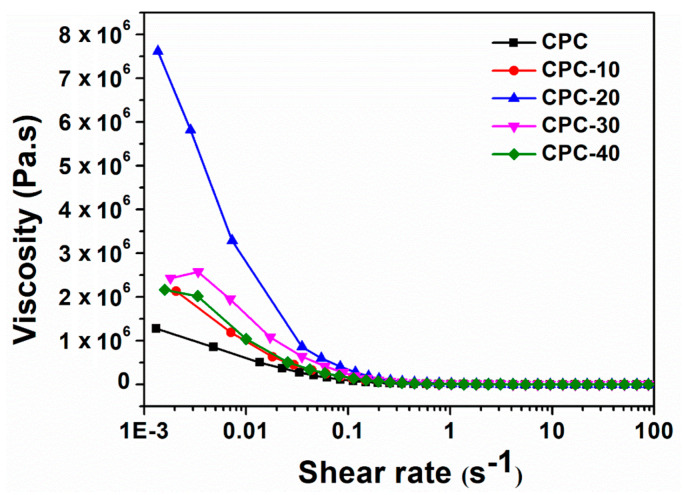
The viscosity versus shear rate behavior of CPC and PLGA-PEG-PLGA/CPCs at 37 °C.

The influence of PLGA-PEG-PLGA hydrogel on the viscosity of CPC paste at 37 °C is shown in Figure 5. It could be seen that the hydrogel greatly improved the viscosity of CPC paste, especially for CPC-20: its viscosity far surpassed the other cement paste at low shear rate. As the shear rate increased, the viscosity of PLGA-PEG-PLGA/CPCs paste decreased sharply, showing shear thinning characteristics like CPC.

### 3.5. Compressive Strength

**Figure 6:**
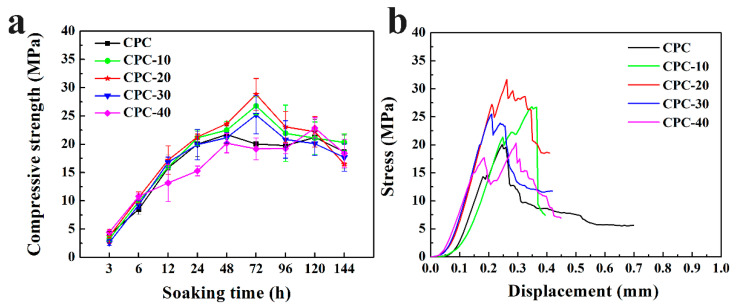
(**a**) The compressive strength of PLGA-PEG-PLGA/CPCs and CPC after immersion for different times; (**b**) stress vs. crosshead displacement curves of sample CPC and PLGA-PEG-PLGA/CPCs soaked for 72 h (n = 4).

Figure 6a exhibits the compressive strength of PLGA-PEG-PLGA/CPCs and CPC after soaking for different time periods. With the prolongation of soaking time, the compressive strength of all samples increased progressively, reaching the highest value at different times, and then exhibited a small decline. Sample CPC-10, CPC-20, CPC-30 reached their highest value at 72 h, while that of sample CPC and CPC-40 was 48 h. The highest compressive strength of sample CPC-20 was about 27 MPa, which was greater than that of other samples. The stress–displacement curves of PLGA-PEG-PLGA/CPCs and CPC after soaking for 72 h are plotted in Figure 6b. The sample CPC-20 was able to withstand the maximum stress of 32 MPa during the uniaxial compressive strength until the fatigue failures happened, whereas the sample CPC only withstood the stress of 20 MPa.

### 3.6. Porosity

**Figure 7:**
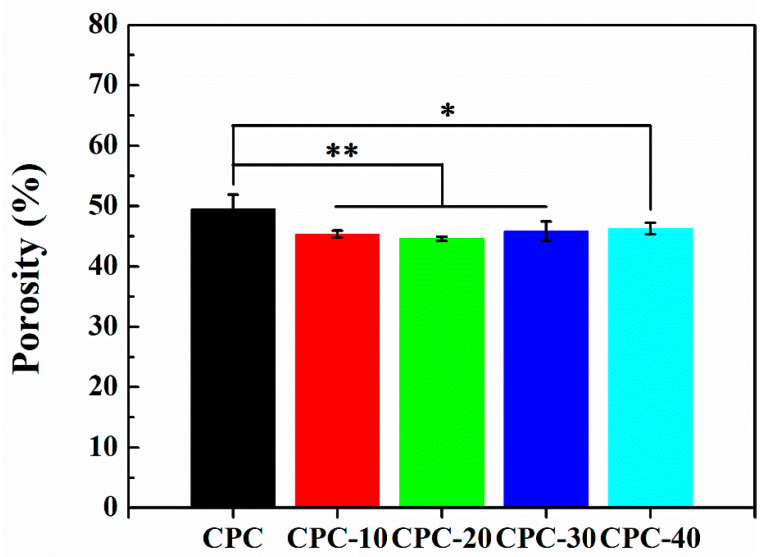
The porosity of CPC and PLGA-PEG-PLGA/CPCs after immersion for three days (n = 5, ** *p* < 0.001, * *p* < 0.05).

Figure 7 shows the influence of PLGA-PEG-PLGA hydrogel on the porosity of CPC. After immersion for three days, the porosity of CPC was 50 ± 2%, while the porosity of CPC-10/20/30/40 was 45 ± 1%, 44 ± 0.5%, 46 ± 2% and 46 ± 1% respectively. It was obvious that the addition of the hydrogel had reduced the porosity, meanwhile there was a significant difference (*p* < 0.05) between the CPC and PLGA-PEG-PLGA/CPCs group.

### 3.7. In Vitro Degradation

#### 3.7.1. Weight Loss

**Figure 8:**
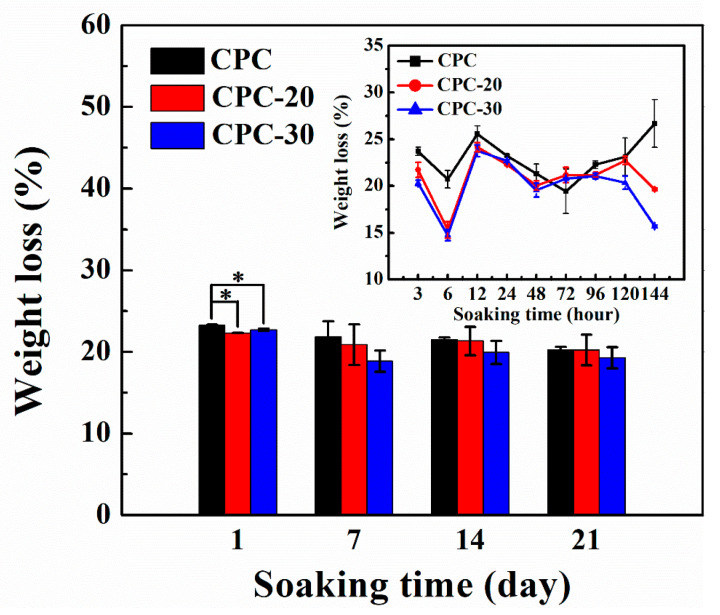
Weight loss% of sample CPC, CPC-20 (20% *v*/*v* hydrogel/CPC composite) and CPC-30 (30% *v*/*v* hydrogel/CPC composite) during degradation studies (n = 4, * *p* < 0.05).

It can be seen from the above results that sample CPC-10 and CPC-40 could not meet operation requirements due to the poor injectability and compressive strength, so only sample CPC-20 and CPC-30 were selected in the following study. The weight loss of PLGA-PEG-PLGA/CPCs and CPC after immersion in SBF solution for certain intervals is shown in Figure 8. Before 144 h, the weight loss of composite CPCs and CPC changed apparently, meanwhile the composite CPCs had lower weight loss than CPC. After seven days, the weight loss of CPC slowed down, whereas the weight loss of composite cements began to increase and the weight loss of sample CPC-20 kept up with that of CPC after 21 days.

##### 3.7.2. pH Value

**Figure 9:**
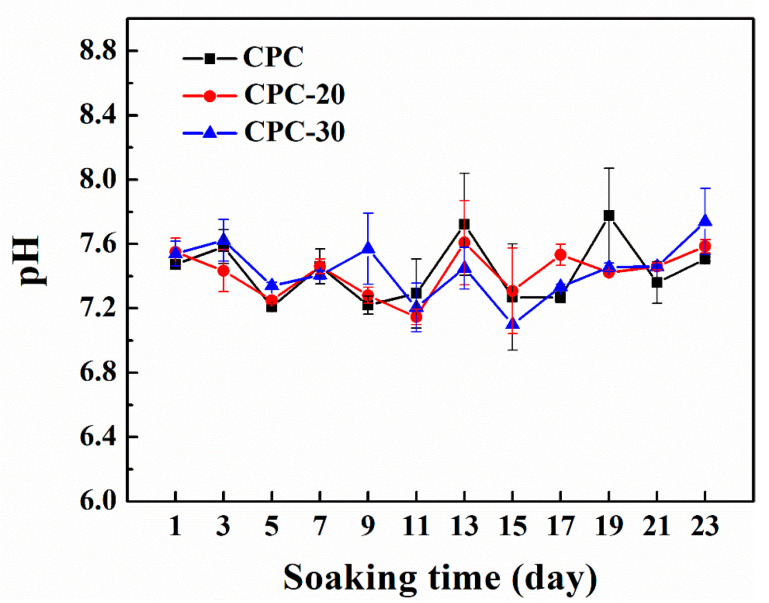
Change in pH value of SBF solution during PLGA-PEG-PLGA/CPCs degradation studies (n = 4).

In order to assess whether PLGA-PEG-PLGA hydrogel may lead to an acidic environment, the samples were immersed in SBF solution and the pH value of SBF solution was shown in Figure 9. Although the composite CPCs displaced a relatively lower pH value compared to CPC, the whole pH value was in a mild range from 7.0 to 7.7, which was close to the pH value of human body fluid.

### 3.8. Phase Composition Analysis

**Figure 10:**
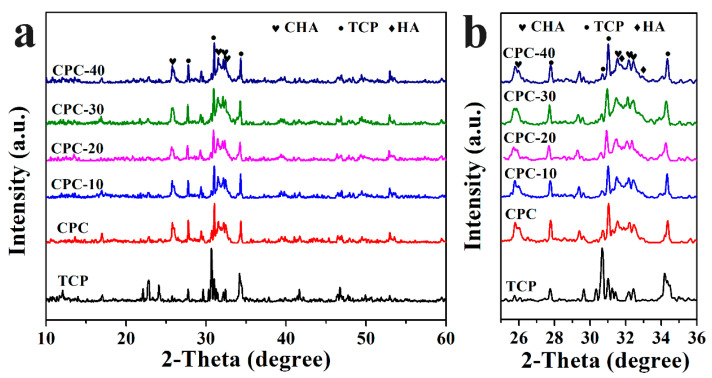
XRD pattern of sample CPC and PLGA-PEG-PLGA/CPCs after immersion for three days (**a**) and enlarged image from 25 to 36 degrees (**b**).

Phase components of PLGA-PEG-PLGA/CPCs and CPC after soaking for three days were shown in Figure 10a,b. The major phases of all samples were carbonate-substituted hydroxyapatite (CHA, PDF#35-0180) and hydroxyapatite (HA, PDF#74-0565), while some residual TCP was also detected (PDF#09-0169). With the volume ratio of hydrogel increasing to 20% *v*/*v*, the relative intensity of HA peaks and CHA peaks increased whilst the intensity of TCP peaks decreased (Figure 10b). As the volume ratio of hydrogel continued to rise to 40% *v*/*v* (CPC-40), the relative intensity of CHA and HA weakened compared with other samples.

### 3.9. FTIR Spectra

**Figure 11:**
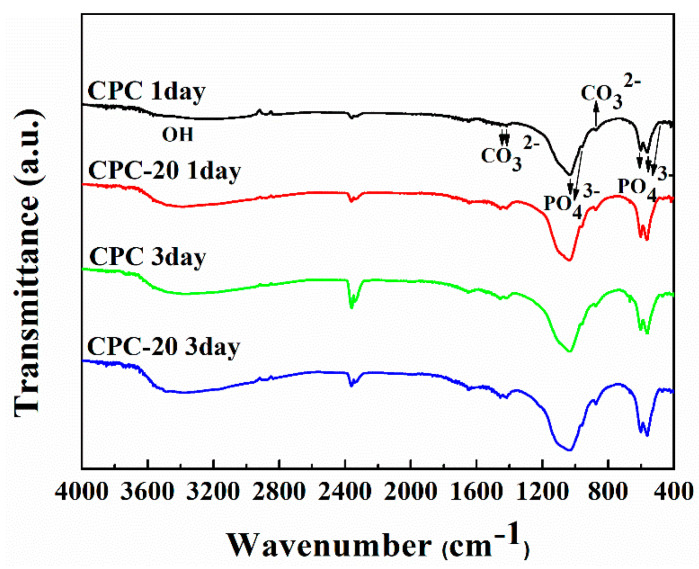
FTIR spectra of sample CPC and CPC-20 after immersion for one day and three days.

Figure 11 showed the FTIR spectra of the sample CPC and CPC-20 after soaking for one day and three days. For sample CPC and CPC-20 soaked for one day, the characteristic bands of phosphate ions appeared at about 1034, 960, 601–561 and 470 cm^−1^, indicating the formation of the HA phase. The double peaks at 1417 and 1456 cm^−1^ as well as the signal at 874 cm^−1^ were attributed to CO_3_^2−^ group of carbonate-substituted hydroxyapatite (CHA). The broad band at about 3365 cm^−1^ was assigned to O–H vibration of water molecules absorbed on HA. With the soaking time increased to three days, the peaks in sample CPC and CPC-20 became more noticeable. In addition, a great difference was that the peaks in sample CPC-20 exhibited stronger absorptions than the corresponding peaks in sample CPC in the same immersion time.

### 3.10. XPS Spectra

**Figure 12:**
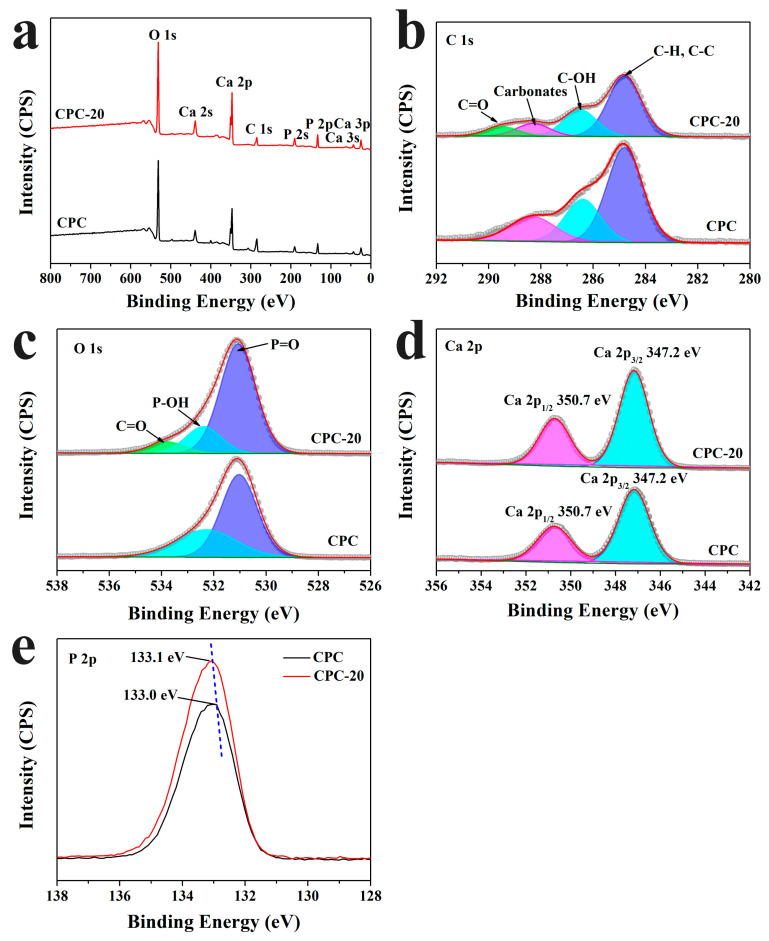
XPS spectra of the surface of CPC and CPC-20 after soaking for three days. (**a**) Wide scan XPS spectra; (**b**) C 1s; (**c**) O 1s; (**d**) Ca 2p; (**e**) P 2p.

For better understanding the chemical state of the elements in sample CPC and CPC-20, XPS analysis was conducted. As shown in Figure 12a, the major constituents of CPC and CPC-20 soaked for three days were oxygen, calcium, carbon and phosphorus. The high-resolution XPS spectra of C1s, O 1s, Ca 2p and P 2p are shown in Figure 12b–e. The C 1s spectrum of CPC could be deconvoluted into three peaks at 284.8 eV, 286.4 eV and 288.3 eV corresponding to the C–H/C–C, C–OH and carbonate-type carbon respectively. Whereas except for the above peaks as the CPC, the C 1s spectrum of CPC-20 (Figure 12b) showed a new peak at about 289.5 eV, which is attributed to the C=O of the ester groups in PLGA-PEG-PLGA hydrogel. The O 1s spectrum of CPC showed two peaks at 531.0 eV and 532.3 eV (Figure 12c). The peak at 531.0 eV was due to the P=O groups from α-TCP and HA, and the latter peak was assigned to the P–OH of HA. In the O 1s spectrum of CPC-20 (Figure 12c), the peaks of P=O and P–OH groups were found at 531.1 eV and 532.4 eV respectively, meanwhile the peak of C=O appeared at 533.8 eV. Figure 12d showed the Ca 2p spectrum of CPC and CPC-20. The Ca 2p_3/2_ and Ca 2p_1/2_ of CPC were located at 347.2 eV and 350.7 eV respectively, while the Ca 2p_3/2_ and Ca 2p_1/2_ of CPC-20 were located at the same position as CPC. The P 2p peaks in sample CPC and CPC-20 were fitted at 133.0 eV and 133.1 eV respectively (Figure 12e), indicating that the state of phosphorus was PO_4_^3−^. Furthermore, the peak of P 2p represented a slight shift toward high energy with the absence of the hydrogel.

### 3.11. Microstructure

**Figure 13:**
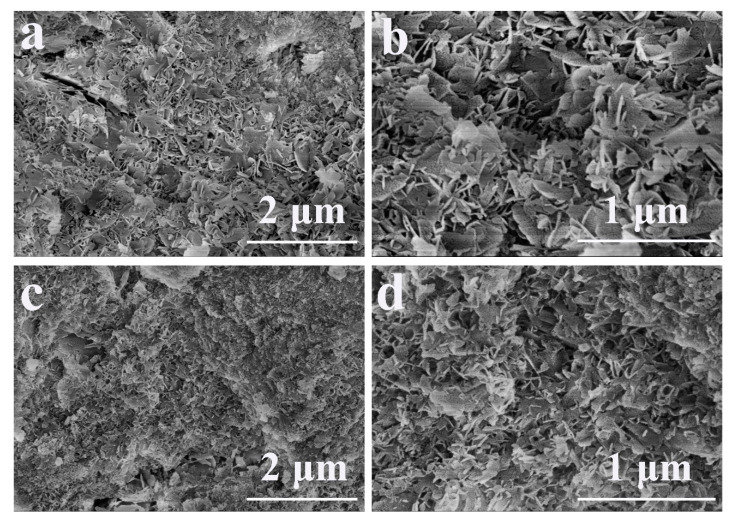
The microstructure of sample CPC after soaking for three days (**a**,**b**) and sample CPC-20 after soaking for three days (**c**,**d**).

The fracture surfaces of sample CPC and CPC-20 after immersion for three days are shown in Figure 13. For sample CPC, flake-like crystals were observed and these crystals entangled with each other, forming a crystal-entangled cement microstructure (Figure 13a,b). In contrast, the size of crystals in sample CPC-20 was smaller than that in CPC (Figure 13c) and sample CPC-20 exhibited a denser microstructure compared to CPC (Figure 13d).

## 4. Discussion

In this study, PLGA-PEG-PLGA hydrogel was introduced to calcium phosphate cement as a constituent of liquid phase to improve the injectability and anti-washout property of CPC, and its effect on the setting characteristics and compressive strength of CPC was investigated by standard testing. The results showed that the various volume ratios of hydrogel obviously improved the injectability and anti-washout property of CPC. Moreover, the PLGA-PEG-PLGA/CPCs showed higher compressive strength than CPC after immersion for 72 h. This finding identified for the first time that a thermosensitive hydrogel could enhance the anti-washout property and simultaneously improve the initial compressive strength of CPC.

**Figure 14:**
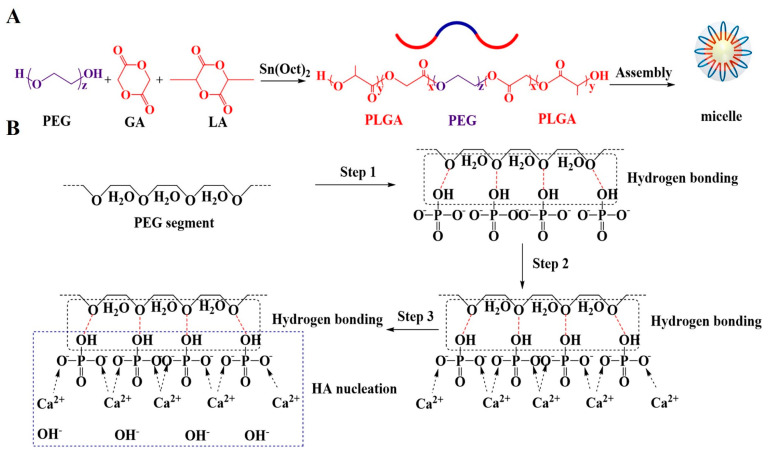
(**A**) The structure of PLGA-PEG-PLGA copolymer. (**B**) Schematic illustration showing the effect of PLGA-PEG-PLGA hydrogel on the hydration process of CPC.

As an injectable bone substitute, the ideal CPCs should have sufficient flowability to be injected through the needle and an excellent anti-washout property to prevent disintegration during implantation [28,29].The most important feature of PLGA-PEG-PLGA hydrogel used in this study was its sol-gel transition at body temperature, which could maintain the composite cement pastes at a low viscosity in vitro and then instantaneously increase the viscosity of pastes when the pastes were injected into the bone fracture site. Before increasing the temperature to 37 °C, the hydrogel self-assembled into micelles to decrease the exposed surface area of hydrophobic PLGA to water molecules, with hydrophilic PEG segments forming loops on the surface and hydrophobic PLGA blocks becoming the cores of the micelles (Figure 14A) [21].The micelles were soluble in an aqueous environment and the hydrogel kept as a sol on account of the hydrogen bonding between hydrophilic PEG segments and water molecules [20]. When uniformly mixing the hydrogel in sol state with CPC paste at room temperature, the hydrogel dispersed in the CPC paste reduced the frictional resistance derived from the rough surfaces of CPC particles and prevented the particles from forming agglomerates, leading to a low viscosity and homogeneous paste. Therefore, after adding 20, 30 and 40% *v*/*v* volume ratio of hydrogel into the liquid phase of CPC, the CPC paste could be easily pushed out from the syringe and the injectability of CPC greatly increased to 72.9% (sample CPC-20) (Figure 3).

As the temperature increased to 37 °C, the hydrogen bonds between PEG and water molecules became weaker and the hydrogel exhibited a sol-gel translation via micellar aggregation and bridging, forming a continuous organic network [30]. This network increased the viscosity of liquid phase rapidly and enhanced the cohesion of CPC matrix (Figure 5). Because of the high viscosity of the liquid phase, the anti-washout property of CPC improved. As shown in Figure 4, the CPC pastes containing hydrogel had fewer fragmentations when they were injected into SBF solution at 37 °C, and sample CPC-20 showed a better anti-washout property than CPC after being shaken for 30 min, which was consistent with the result of viscosity test.

Setting time is of great importance for evaluating the clinical perspective of calcium phosphate cement [31]. Ideally, the initial setting time should be approximately 15 min, the final setting time should under 30 min [31,32]. After adding different volume ratios of hydrogel into the setting liquid of CPC, both initial and final setting time had been prolonged, only the setting time of sample CPC-20 was slightly longer than that of sample CPC and acceptable for clinical use, which was about 15 min and 30 min respectively (Figure 2). The hypothesis that water-soluble polymer with higher hydrophilicity than calcium phosphate (CaP) ceramics would increase the setting time had been reported by previous works [33,34]. Because of the hydrogen bonding between PEG segments and water molecules, the PEG segments possess higher hydrophilicity than CPC particles and extract water from the cement. This high hydrophilicity of PEG segments hindered the dissolution of CPC particles, resulting in an increase of setting time. The difference of setting time among CPC-20/30/40 was mainly due to the existence of a large number of PEG segments in CPC-30 and CPC-40. Due to the existence of a large number of PEG segments, CPC-30 and CPC-40 exhibited a relatively longer final setting time compared with CPC-20.

The early mechanical strength of CPC is a vital aspect requiring more attention that determines the success or failure of the early-stage implant [35]. It is well known that the compressive strength of CPC is related to the hydration reaction of CPC particles, during which the newly formed hydration product precipitated on the surface of the particles and continued to grow to needle-like or flake-like crystals with the progress of the reaction [36,37]. These crystals entangled with each other and made the microstructure of CPC more compact, which would further increase the compressive strength of CPC [38]. Unlike other biopolymers that produced an unfavorable effect on the early strength of CPC, the incorporation of hydrogel had improved the initial compressive strength of the cement (Figure 6). It was worth knowing the effect of hydrogel on the hydration reaction and microstructure of CPC.

Based on the result of XRD, the PLGA-PEG-PLGA hydrogel had influence the phase translation of CPC. As shown in Figure 10a,b, the relative diffraction intensity of HA in PLGA-PEG-PLGA/CPCs (except CPC-40) was higher than that in sample CPC, indicating that the content of HA formed in PLGA-PEG-PLGA/CPCs (except CPC-40) was higher than that formed in CPC. The strong absorptions of typical HA peaks in the FTIR spectra of sample CPC-20 (Figure 11) was in accordance with the XRD results. In the O 1s spectrum of CPC-20 (Figure 12c), the binding energy of the P-OH peak which belonged to HA was 0.1 eV higher than that of the corresponding peak in CPC, confirming that the hydrogel influenced the formation of HA. In the other words, the addition of 20% *v*/*v* hydrogel promoted the formation of HA, which accounted for the better compressive strength of PLGA-PEG-PLGA/CPCs as compared to CPC (Figure 6).

The positive effect of PLGA-PEG-PLGA hydrogel on the formation of HA was mainly from the PEG segments which existed on the shells of the PLGA-PEG-PLGA micelles (Figure 14A). As determined by XPS (Figure 12), the binding energies of P 2p (Figure 12e) in CPC-20 moved to high energy compared with the corresponding P 2p peaks in CPC, proving that the hydrogel changed the chemical state of phosphorus in the hydrated cement. A possible process of the hydration reaction of CPC with absence of PEG segments was shown in Figure 14B. The bridging oxygen atom in the hydrophilic PEG segment formed hydrogen bonding with the hydroxyl group in the hydrogen phosphate ions (Step 1). Then, the calcium ions were attracted to the hydrogen phosphate ions by electrostatic force (Step 2). Finally, the calcium ions interacted with the hydroxyl ions in the SBF solution to the nucleation of HA as following Equation (4) [39] (Step 3):

10Ca^2+^ + 6HPO_4_^2−^ + 8OH^−^→Ca_10_ (PO_4_)_6_ (OH)_2_ + 6H_2_O (4)

In short, the PEG segments on the surface of the micelles accelerated the nucleation of HA via establishing hydrogen bonds with HPO_4_^2−^.

Due to the facilitation from the PEG segments of the hydrogel, numerous HA nucleated and grew to flake-like crystals with fine grain size, leading to a reduction of porosity (Figure 7) and a compact microstructure of CPC-20 (Figure 13c, d). According to the Hall–Petch effect, the yield strength of materials is inversely proportional to its crystal size [40]. Meanwhile, the reduction of porosity was beneficial for the mechanical properties of CPC [41]. Therefore, the compressive strength of sample CPC-20 was higher than that of CPC after immersion for three days. The positive effect of PEG segment on the nucleation of HA also contributed to the compressive strength of CPC-30 and CPC-40. Nevertheless, the hydrogel network formed in CPC-30 and 40 was too thick to maintain the compressive strength at high level, considering that the hydrogel network as the second phase in the CPC matrix might destroy the entanglement network of HA crystals.

From the above results, adding appropriate volume ratios of hydrogel into the liquid phase could obviously improve the injectability and anti-washout property of CPC, as well as having the high strength and suitable setting time for surgery. It is necessary to understand the degradation behavior of the composite cements. It was well known that the degradation rate of CPC is too poor to match the rate of new bone formation [42]. As shown in Figure 8, although the weight loss of CPC-20 and CPC-30 was lower than that of CPC before seven days, the degradation rate of CPC-20 had increased and kept up with that of CPC after 21 days, which mainly stemmed from the resorption of CHA and the progressive hydrolyzation of the hydrogel. As shown in the XRD pattern (Figure 10), the CHA was also detected in the hydrated cements. During the formation of HA, the carbonate ions might substitute the hydroxy ions of HA according to Equation (5) [43], taking into account that the CaCO_3_ was contained in the CPC particles and carbon dioxide gas was present in the SBF solution.

Ca_10_ (PO_4_)_6_ (OH)_2_ + CO_2_→Ca_10_ (PO_4_)_6_ (OH)_(2−X)_ (CO_3_)_X_ + H_2_O (5)

The CHA was considered as an optimized biomaterial because it showed better resorption in vivo than stoichiometric HA and had the ability to increase the osteoclastogenesis, which was beneficial for the faster bonding between the implantation materials and human bone [44]. Owing to the high content of soluble CHA in the CPC-20, the weight loss of CPC-20 progressively increased after three days and their compressive strength showed a decline after immersion for 96 h (Figure 6a). On the other hand, the hydrolyzation of PLGA-PEG-PLGA hydrogel also contributed to the weight loss of CPC-20. The hydrolyzation of PLGA copolymer was accompanied by the production of lactic and glycolic acid, which could accelerate the degradation of apatite calcium phosphate cement [45]. As shown in Figure 9, the pH of SBF solution where the CPC-20 and CPC-30 was incubated was relatively lower than that of SBF solution where CPC was incubated, proving the presence of acid byproducts. However, the hydrolyzation of hydrogel did not produce a strongly acidic environment.

In summary, considering the setting characteristics, compressive strength and degradation behavior of PLGA-PEG-PLGA/CPCs, 20% *v*/*v* of hydrogel was the optimum volume ratio for the modification of calcium phosphate cement. Furthermore, the particle size of the solid phase could be optimized to reduce the setting time of CPC-20. Meanwhile, more in vitro cytocompatibility assessment and in vivo study should be performed to evaluate the potential application of PLGA-PEG-PLGA/CPCs.

## 5. Conclusions

In this study, an injectable calcium phosphate cement composite was successfully fabricated by combined with PLGA-PEG-PLGA hydrogel. The hydrogel improved the injectability and anti-washout property of CPC paste by its sol-gel translation, but meanwhile prolonged the setting time of CPC because of the hydrophilicity of PEG segments. When the volume ratio of hydrogel in the liquid phase was 20% *v*/*v*, the composite cement had suitable initial and final setting time, which was 15 min and 30 min respectively. Furthermore, the compressive strength of composite cement with 20% *v*/*v* hydrogel reached 27 MPa, which was higher than that of CPC and other composite cements. The high compressive strength was attributed to the positive effect of hydrogel on the formation of hydroxyapatite, which led to a compact structure of hydrated cement. In vitro degradation showed that the hydrogel had no negative affect on the overall degradation rate of CPC. Overall, the PLGA-PEG-PLGA hydrogel could be a potential additive to modify the handling properties and compressive strength of CPC.

## Figures and Tables

**Table 1:** Composition and physicochemical properties of other studies concerning CPC.

Solid Phase	Liquid Phase	Setting Time (min)	Compressive Strength (MPa)	Injectability /Anti-Washout Property	Ref.
Partially crystallized calcium phosphate (PCCP), CaHPO_4_ (DCPA), HA, PAAS	Deionized water	T_i_ = 11 T_f_ = 30	23.4	yes/well	[10]
CaHPO_4_, Tetracalcium phosphate (TTCP), gelatin microspheres	6 wt% Na_2_HPO_4_ solution	T_i_ = 14 T_f_ = 25	6.5	yes/not researched	[11]
PCCP, DCPA	KGM/GG solution	T_i_ = 10 T_f_ = 20	15	yes/well	[12]
PCCP, DCPA	1% LBG solution	T_i_ = 16 T_f_ = 40	15.9	yes/well	[13]
Alpha tricalcium phosphate (α-TCP), CaCO_3_, HA	2% Chondroitin Sulfate solution	T_i_ = 7 T_f_ = 53	22	high/poor	[14]

**Table 2:** Composition of the setting liquid for preparing the composite cement.

Samples	Na_2_HPO_4_, % *v/v*	PLGA-PEG-PLGA Hydrogel, % *v/v*
CPC	100	0
CPC-10	90	10
CPC-20	80	20
CPC-30	70	30
CPC-40	60	40

## References

[1] Shi H., Zhang W., Liu X., Zeng S., Yu T., Zhou C. (2019). Synergistic effects of citric acid—Sodium alginate on physicochemical properties of α-tricalcium phosphate bone cement. Ceram. Int..

[2] Sahin E., Kalyon D.M. (2017). The rheological behavior of a fast-setting calcium phosphate bone cement and its dependence on deformation conditions. J. Mech. Behav. Biomed. Mater..

[3] Paknahad A., Kucko N.W., Leeuwenburgh S.C.G., Sluys L.J. (2020). Experimental and numerical analysis on bending and tensile failure behavior of calcium phosphate cements. J. Mech. Behav. Biomed. Mater..

[4] Castro A.G.B., Polini A., Azami Z., Leeuwenburgh S.C.G., Jansen J.A., Yang F., van den Beucken J.J.J.P. (2017). Incorporation of PLLA micro-fillers for mechanical reinforcement of calcium-phosphate cement. J. Mech. Behav. Biomed. Mater..

[5] Shu Y., Zhou Y., Ma P., Li C., Ge C., Wang Y., Li Q., Yu K., Lu R., Zou X. (2019). Degradation in vitro and in vivo of beta-TCP/MCPM-based premixed calcium phosphate cement. J. Mech. Behav. Biomed. Mater..

[6] Bruckner T., Meininger M., Groll J., Kubler A.C., Gbureck U. (2019). Magnesium Phosphate Cement as Mineral Bone Adhesive. Materials.

[7] No Y.J., Holzmeister I., Lu Z.F., Prajapati S., Shi J., Gbureck U., Zreiqat H. (2019). Effect of Baghdadite Substitution on the Physicochemical Properties of Brushite Cements. Materials.

[8] Torres P.M., Gouveia S., Olhero S., Kaushal A., Ferreira J.M. (2015). Injectability of calcium phosphate pastes: Effects of particle size and state of aggregation of beta-tricalcium phosphate powders. Acta Biomater..

[9] Liu C.S., Shao H.F., Chen F.Y., Zheng H.Y. (2006). Rheological properties of concentrated aqueous injectable calcium phosphate cement slurry. Biomaterials.

[10] Li X.M., He F.P., Ye J.D. (2017). Preparation, characterization and in vitro cell performance of anti-washout calcium phosphate cement modified by sodium polyacrylate. RSC Adv..

[11] Nezafati N., Farokhi M., Heydari M., Hesaraki S., Nasab N.A. (2019). In vitro bioactivity and cytocompatablity of an injectable calcium phosphate cement/silanated gelatin microsphere composite bone cement. Compos. B Eng..

[12] Qian G., Li X., He F., Ye J. (2018). Improvement of anti-washout property of calcium phosphate cement by addition of konjac glucomannan and guar gum. J. Mater. Sci. Mater. Med..

[13] Liu J.Q., Li J.Y., Ye J.D. (2016). Properties and Cytocompatibility of Anti-Washout Calcium Phosphate Cement by Introducing Locust Bean Gum. J. Mater. Sci. Technol..

[14] Shi H., Ye X., Zhang J., Ye J. (2018). Enhanced Osteogenesis of Injectable Calcium Phosphate Bone Cement Mediated by Loading Chondroitin Sulfate. ACS Biomater. Sci. Eng..

[15] Liu H., Zhang Z., Gao C., Bai Y., Liu B., Wang W., Ma Y., Saijilafu, Yang H., Li Y. (2020). Enhancing effects of radiopaque agent BaSO4 on mechanical and biocompatibility properties of injectable calcium phosphate composite cement. Mater. Sci. Eng. C.

[16] Wang S., Xu C., Yu S.C., Wu X.P., Jie Z., Dai H.L. (2019). Citric acid enhances the physical properties, cytocompatibility and osteogenesis of magnesium calcium phosphate cement. J. Mech. Behav. Biomed. Mater..

[17] Hettich G., Schierjott R.A., Epple M., Gbureck U., Heinemann S., Mozaffari-Jovein H., Grupp T.M. (2019). Calcium Phosphate Bone Graft Substitutes with High Mechanical Load Capacity and High Degree of Interconnecting Porosity. Materials.

[18] Weichhold J., Gbureck U., Goetz-Neunhoeffer F., Hurle K. (2019). Setting Mechanism of a CDHA Forming alpha-TCP Cement Modified with Sodium Phytate for Improved Injectability. Materials.

[19] Maazouz Y., Montufar E.B., Malbert J., Espanol M., Ginebra M.P. (2017). Self-hardening and thermoresponsive alpha tricalcium phosphate/pluronic pastes. Acta Biomater..

[20] Sun J.G., Liu X., Lei Y., Tang M.Y., Dai Z.X., Yang X.W., Yu X.B., Yu L., Sun X.H., Ding J.D. (2017). Sustained subconjunctival delivery of cyclosporine A using thermogelling polymers for glaucoma filtration surgery. J. Mat. Chem. B.

[21] Osorno L.L., Maldonado D.E., Whitener R.J., Brandley A.N., Yiantsos A., Medina J.D.R., Byrne M.E. (2020). Amphiphilic PLGA-PEG-PLGA triblock copolymer nanogels varying in gelation temperature and modulus for the extended and controlled release of hyaluronic acid. J. Appl. Polym. Sci..

[22] Lai M.C., Chang K.C., Hsu S.C., Chou M.C., Hung W.I., Hsiao Y.R., Lee H.M., Hsieh M.F., Yeh J.M. (2014). In situ gelation of PEG-PLGA-PEG hydrogels containing high loading of hydroxyapatite: In vitro and in vivo characteristics. Biomed. Mater..

[23] Peng K.T., Chen C.F., Chu I.M., Li Y.M., Hsu W.H., Hsu R.W., Chang P.J. (2010). Treatment of osteomyelitis with teicoplanin-encapsulated biodegradable thermosensitive hydrogel nanoparticles. Biomaterials.

[24] Yu L., Xu W., Shen W.J., Cao L.P., Liu Y., Li Z.S., Ding J.D. (2014). Poly(lactic acid-co-glycolic acid)-poly(ethylene glycol)-poly(lactic acid-co-glycolic acid) thermogel as a novel submucosal cushion for endoscopic submucosal dissection. Acta Biomater..

[25] Ding Z.W., Li H., Wei J., Li R.J., Yan Y.G. (2018). Developing a novel magnesium glycerophosphate/silicate-based organic-inorganic composite cement for bone repair. Mater. Sci. Eng. C Mater. Biol. Appl..

[26] Liu W.Z., Zhang J.T., Weiss P., Tancret F., Bouler J.M. (2013). The influence of different cellulose ethers on both the handling and mechanical properties of calcium phosphate cements for bone substitution. Acta Biomater..

[27] Wu I.T., Kao P.F., Huang Y.R., Ding S.J. (2020). In vitro and in vivo osteogenesis of gelatin-modified calcium silicate cement with washout resistance. Mater. Sci. Eng. C Mater. Biol. Appl..

[28] Habraken W.J., Liao H.B., Zhang Z., Wolke J.G., Grijpma D.W., Mikos A.G., Feijen J., Jansen J.A. (2010). In vivo degradation of calcium phosphate cement incorporated into biodegradable microspheres. Acta Biomater..

[29] Zhang J., Liu W., Schnitzler V., Tancret F., Bouler J.M. (2014). Calcium phosphate cements for bone substitution: Chemistry, handling and mechanical properties. Acta Biomater..

[30] Kamali H., Khodaverdi E., Hadizadeh F. (2018). Ring-opening polymerization of PLGA-PEG-PLGA triblock copolymer in supercritical carbon dioxide. J. Supercrit. Fluids.

[31] Smith B.T., Lu A., Watson E., Santoro M., Melchiorri A.J., Grosfeld E.C., van den Beucken J., Jansen J.A., Scott D.W., Fisher J.P. (2018). Incorporation of fast dissolving glucose porogens and poly(lactic-co-glycolic acid) microparticles within calcium phosphate cements for bone tissue regeneration. Acta Biomater..

[32] Sugawara A., Asaoka K., Ding S.J. (2013). Calcium phosphate-based cements: Clinical needs and recent progress. J. Mat. Chem. B.

[33] Lodoso-Torrecilla I., Stumpel F., Jansen J.A., van den Beucken J. (2020). Early-stage macroporosity enhancement in calcium phosphate cements by inclusion of poly(N-vinylpyrrolidone) particles as a porogen. Mater. Today Commun..

[34] Smith B.T., Santoro M., Grosfeld E.C., Shah S.R., van den Beucken J.J.J.P., Jansen J.A., Mikos A.G. (2017). Incorporation of fast dissolving glucose porogens into an injectable calcium phosphate cement for bone tissue engineering. Acta Biomater..

[35] Dorozhkin S.V. (2008). Calcium orthophosphate cements for biomedical application. J. Mater. Sci.

[36] Yu T., Gao C.Y., Ye J.D., Zhang M. (2014). Synthesis and Characterization of a Novel Silver-Substituted Calcium Phosphate Cement. J. Mater. Sci. Technol..

[37] Richter R.F., Ahlfeld T., Gelinsky M., Lode A. (2019). Development and Characterization of Composites Consisting of Calcium Phosphate Cements and Mesoporous Bioactive Glass for Extrusion-Based Fabrication. Materials.

[38] Yao Z.Q., Ivanisenko Y., Diemant T., Caron A., Chuvilin A., Jiang J.Z., Valiev R.Z., Qi M., Fecht H.J. (2010). Synthesis and properties of hydroxyapatite-containing porous titania coating on ultrafine-grained titanium by micro-arc oxidation. Acta Biomater..

[39] Chen X.B., Li Y.C., Hodgson P.D., Wen C. (2009). Microstructures and bond strengths of the calcium phosphate coatings formed on titanium from different simulated body fluids. Mat. Sci. Eng. C Bio. S.

[40] Koh I., Lopez A., Pinar A.B., Helgason B., Ferguson S.J. (2015). The effect of water on the mechanical properties of soluble and insoluble ceramic cements. J. Mech. Behav. Biomed. Mater..

[41] Liu W., Zhang J., Rethore G., Khairoun K., Pilet P., Tancret F., Bouler J.M., Weiss P. (2014). A novel injectable, cohesive and toughened Si-HPMC (silanized-hydroxypropyl methylcellulose) composite calcium phosphate cement for bone substitution. Acta Biomater..

[42] Nie L., Chen D., Fu J., Yang S.H., Hou R.X., Suo J.P. (2015). Macroporous biphasic calcium phosphate scaffolds reinforced by poly-L-lactic acid/hydroxyapatite nanocomposite coatings for bone regeneration. Biochem. Eng. J..

[43] Chaudhry A.A., Knowles J.C., Rehman I., Darr J.A. (2013). Rapid hydrothermal flow synthesis and characterisation of carbonate- and silicate-substituted calcium phosphates. J. Biomater. Appl..

[44] Spence G., Patel N., Brooks R., Bonfield W., Rushton N. (2010). Osteoclastogenesis on hydroxyapatite ceramics: The effect of carbonate substitution. J. Biomed. Mater. Res. A.

[45] Lanao R.P.F., Leeuwenburgh S.C.G., Wolke J.G.C., Jansen J.A. (2011). In vitro degradation rate of apatitic calcium phosphate cement with incorporated PLGA microspheres. Acta Biomater..

